# Early rising asexual parasitaemia in Nigerian children following a first dose of artemisinin-based combination treatments of falciparum malaria

**DOI:** 10.1186/s12879-016-2173-z

**Published:** 2017-01-31

**Authors:** Akintunde Sowunmi, Kazeem Akano, Adejumoke I. Ayede, Elsie O. Adewoye, Godwin Ntadom, Bayo Fatunmbi, Grace O. Gbotosho, Onikepe A. Folarin, Christian T. Happi

**Affiliations:** 1 Department of Pharmacology and Therapeutics, University of Ibadan, Ibadan, Nigeria; 2 Institute for Medical Research and Training, University of Ibadan, Ibadan, Nigeria; 3 Department of Paediatrics, University of Ibadan, Ibadan, Nigeria; 4 Department of Physiology, University of Ibadan, Ibadan, Nigeria; 5 National Malaria Elimination Programme, Federal Ministry of Health, Abuja, Nigeria; 6 World Health Organization, Regional Office for the Western Pacific, Phnom Penh, Cambodia; 7 Department of Biological Sciences, Redeemer’s University, Ede, Nigeria; 8 African Centre of Excellence for Genomics of Infectious Diseases (ACEGID), Redeemer’s University, Ede, Nigeria; 9 Department of Clinical Pharmacology, University College Hospital, Ibadan, Nigeria

**Keywords:** Early rising asexual parasitaemia, Artemisinin-based combination treatments, Children, Nigeria

## Abstract

**Background:**

Early rising asexual parasitaemia (ERAP), initially defined as ‘an increase in the parasite count over the baseline pre-treatment level during the first 24 h of treatment’ of falciparum malaria with artemisinin derivatives is well documented, but there is no characterization of its risk factors, kinetics, molecular features or relationship to late-appearing anaemia (LAA) in acute falciparum malaria in African children following oral artemisinin-based combination therapies (ACTs).

**Methods:**

ERAP was defined as ≥5% increase in pre-treatment parasitaemia within 8 h of initiating treatment. Parasitaemia was quantified pre-treatment and 1–2 hourly for 8 h, and less frequently thereafter for 6 weeks following randomized treatment of acutely malarious children with artesunate-amodiaquine, artemether-lumefantrine or dihydroartemisinin-piperaquine. Risk factors were determined by stepwise multiple logistic regression model. Kinetics of release into and of elimination of asexual parasites and DNA clones from peripheral blood were evaluated by method of residuals and non-compartment model, respectively. Parasite population changes were evaluated morphologically and by molecular genotyping.

**Results:**

ERAP occurred in 205 of 416 children. A parasitaemia <100,000/μL and parasitaemia 1 day post-treatment initiation were independent predictors of ERAP. In children with ERAP: mean and peak time of increase in parasitaemia were 105.6% (95% CI 81–130.1) and 2.5 h (95% CI 2.2–2.7), respectively. Mean lag time, half-time and rate constant of release were 0.2 h (95% CI 0.2–0.3), 1 h (95% CI 0.9–1.1), and 0.9 h^−1^ (95% CI 0.8–1), respectively. Schizonts and young gametocytes were seen only in peripheral blood of few children with ERAP. In age-, gender-, baseline parasitaemia- and treatment-matched children with and without ERAP, parasite DNA clearance time and area under curve of number of DNA clones *versus* time were significantly higher in children with ERAP indicating peripheral retention of released parasites followed by elimination. DNA clone elimination was monoexponential.

**Conclusion:**

ERAP is common, occurs rapidly as first order process and may be due to mobilization of parasites from deep tissue following a first dose of ACTs of acute childhood falciparum malaria.

**Trials registration:**

Pan African Clinical Trial Registry PACTR201508001188143, 3 July 2015; PACTR201510001189370, 3 July 2015; PACTR201508001191898, 7 July 2015 and PACTR201508001193368, 8 July 2015.

## Background

Early rising asexual parasitaemia (ERAP), originally described as an increase in parasite count over the baseline pre-treatment level occurring within 24 h following treatment of falciparum malaria with artemisinin-like drugs [1], may occur in 25–45% of malarious patients following artemisinin-based combination treatments (ACTs) [1–3]. Although prognosis is favourable in malarious Thai and Nigerian patients with ERAP [1–3], the factors contributing to ERAP, and the additional burden posed by it are little evaluated. Given that early increases in parasitaemia may be accompanied by changes in asexual and sexual parasite populations following ACTs and may be relatively short-lasting by morphological assessment [3], evaluation of ERAP by polymerase chain reaction (PCR) genotyping and kinetics of parasite population changes are urgently needed as they may provide insight into the possible cause(s) and consequences of ERAP and the possible mechanisms of ACTs-related ERAP.

Of the estimated 214 million clinical episodes of malaria reported annually, 188 million clinical episodes occur in Africa mainly in children [4]. In Nigeria, an estimated over 37 million clinical episodes of falciparum malaria are reported annually [5]. Although acute falciparum infections are associated with anaemia in 25–80% of African children at presentation [6–8], it is increasingly apparent additional burden of anaemia may be imposed by a late-appearing anaemia (LAA) following successful ACTs of apparently uncomplicated infections [7, 9, 10]. The extent of this additional burden and its relationship to ERAP, if any, following ACTs in African children remain unknown.

In order to determine the factors contributing to ERAP, its release and disposition kinetics, its consequences and other additional burden it may pose, and the association between ERAP and LAA, larger number of well-characterized patients and comparison with patients without ERAP are needed. For these reasons, we added asexual parasite kinetic analyses and molecular genotyping to the clinical and parasitological characterization of children with ERAP following a first dose of ACTs of apparently uncomplicated *Plasmodium falciparum* malaria in children. The aims of the study were to determine in malarious children with ERAP: its frequency and the factors contributing to it; its consequences; the kinetics of release into and of the disposition of asexual stage parasites from peripheral blood during ERAP; asexual stage and gametocyte stage population changes; asexual stage genotyping during ERAP; and the relationship, if any, between ERAP and LAA following ACTs.

## Methods

### Study location

The study was conducted between January 2008 and December 2015 in Ibadan, an endemic area of malaria in southwestern Nigeria. It was part of a larger and longer study of the efficacies of artemisinin-based combination treatments - artesunate-amodiaquine (AA), artemether-lumefantrine (AL) and dihydroartemisinin-piperaquine (DHP). Details of the study have been described elsewhere [7]. The study protocol was approved by The Ethics Committee of The Ministry of Health, Ibadan and by National Health Research Ethics Committee, Abuja, Nigeria [Pan African Clinical Trial Registry PACTR201508001188143; PACTR201510001189370; PACTR201508001191898; PACTR201508001193368].

### Patients

#### Inclusion and exclusion criteria

Patients were enrolled in the study if they met the following criteria: age 6 months–15 years, symptoms compatible with acute uncomplicated malaria with *Plasmodium falciparum* mono-infections ≥2000 μL^−1^ of blood, no history of antimalarial drug ingestion in the 2 weeks prior to enrolment, absence of severe malaria [11–13], written informed consent given by parents or guardians and ability to comply with a 42-day follow-up period. Patients with uncomplicated hyperparasitaemia (parasitaemia >250,000 μL^−1^) were not excluded from the study [13]. Patients with severe malnutrition (i. e. weight for age < 60% and bilateral oedema) and those with sickle cell anaemia were excluded from the study. Patient selection and enrolment were done by a physician who did not participate in patient evaluation once treatment began [7].

#### Drug treatment

Patients were randomised to and received artesunate-amodiaquine, artemether-lumefantrine or dihydroartemisinin-piperaquine orally as previously described [7] (Table 1). Each randomization envelope was opened at the time of treatment by the attending nurse or physician. Each tablet of artemether-lumefantrine (Coartem®, Novatis, Basel, Switzerland) contains 20 mg of artemether and 120 mg of lumefantrine. Each tablet of dihydroartemisinin-piperaquine (Duo-cotecxin®, Zhejiang Holley Nanhu, China) contains 40 mg of dihydroartemisinin and 320 mg of piperaquine. The formulations of artesunate-amodiaquine (Coarsucam®, Sanofi Aventis, France) are 25 mg/67.5 mg, 50 mg/135 mg, 100 mg/270 mg of fixed dose combination. All drugs were given within 3 min of obtaining enrolment (pre-treatment, baseline) blood slides for quantification of parasitaemia.

**Table 1:** Treatment regimens

Treatment regimens
Artemether-lumefantrine Patients weighing: 5–14 kg received 1 tablet 15–24 kg received 2 tablets 25–34 kg received 3 tablets >34 kg received 4 tablets, at presentation (0 h), 8 h later and at 24, 36, 48 and 60 h after the first dose.
Artesunate-amodiaquine Patients weighing: ≥4.5- < 9 kg received 1 tablet of 25 mg/67.5 mg formulation ≥9- < 18 kg received 1 tablet of 50 mg/135 mg formulation ≥18- < 24 kg received 1 tablet of 100 mg/270 mg formulation 24–36 kg received 1.5 tablet of 100 mg/270 mg formulation >36 kg received 2 tablets of 100/270 mg formulation, daily for 3 days.
Dihydroartemisinin-piperaquine Patients weighing: ≥4.5 - <10 kg received ¾ of 1 tablet 10 - <16 kg, received 1.5 tablet 16 - <24 kg, received 2 tablets 24 - <34 or received 2.5 tablets 34 - <50 kg received 3 tablets, daily for 3 days.

#### Patient evaluation

Clinical evaluation and monitoring for adverse events were done at the following times: before treatment (day 0; that is, the day treatment began) and at 1, 2, 3, 7, 14, 21, 28, 35 and 42 days after start of treatment. Clinical evaluation consisted of physical examination and measurement of body temperature, heart and respiratory rates. Side effects were defined as symptoms and signs that first occurred or became worse after treatment started and were checked for at every visit. Any new events occurring during treatment were also considered as side effects.

#### Parasitological evaluation

Thick and thin blood films prepared from a finger prick were obtained at the following times: before treatment (0 h), and at 1, 2, 4, 6, 8, 24, 48, 72, 96, 120, 144, 168, 336, 504 and 672 h and then on days 35 and 42 after initiation of treatment. Blood slides were stained with Giemsa and examined by light microscopy under oil immersion objective lens at 1000 × magnification by two assessors who did not know the drug regimen of the patients. A senior member of the study team reviewed the slides if there was any disagreement between the two microscopists. In addition, the slide of every fourth child enrolled in the study was reviewed by the senior member.

Parasitaemias, asexual or sexual, in thick films were estimated by counting asexual and sexual parasites relative to 500 leukocytes, or 500 asexual or sexual forms whichever occurred first. From this figure, the parasite density was calculated assuming a leukocyte count of 6,000 μL^−1^ of blood [14–16]. A slide was considered parasite negative if no asexual or sexual parasite was detected after examination of 200 microscope fields.

#### Definition of ERAP

ERAP was defined as ≥5% increase in pre-treatment asexual parasitaemia occurring within 8 h of initiating ACTs.

#### Staging of asexual and sexual parasites development in peripheral blood

Stages of asexual parasite development in peripheral blood were estimated as follows: R1: width of cytoplasm/diameter of nucleus <0.5, that is, ring form aged 0- < 6 h. R2: width of cytoplasm/diameter of nucleus >0.5- < 1, that is, ring form aged 6- < 18 h. R3: width of cytoplasm/diameter of nucleus >1, that is, ring form aged 18–24 h. Late trophozoite, that is aged >24–40 h and containing 2 nuclei. Schizonts, that is, >40 h and containing at least 3 nuclei [17]. Gametocytes were sexed according to the following morphological criteria: males (microgametocytes) are smaller than females (macrogametocytes), the nucleus is larger in males than in females, the end of the cells are rounder in males and pointed in females, with Giemsa the cytoplasm stains purple in males and deep blue in females, and the granules of malaria pigment are centrally located in females and more widely spread scattered in males [18, 19]. Gametocytes were classified morphologically as male or female if at least three of the five criteria stated above were present. Gametocytes were considered immature or young when they are Stage I–III, or mature when they are Stage IV–V [20]. Gametocytes were classified as stage II if they were elongated in the erythrocytes or had a D-shape and were distinguished from late trophozoite with 2 nuclei. Gametocytes sex ratio, the proportion of gametocytes that is male [21, 22], was not evaluated.

#### PCR genotyping

PCR genotyping was done as described previously [23, 24]. *P. falciparum* loci that exhibited repeated numbers of polymorphisms to distinguish between different parasite populations were used for characterization of population structure. Block 2 of merozoite surface protein-1 (MSP-1), block 3 of MSP-2, and region II of glutamine-rich protein (GLURP) were amplified by nested polymerase chain reaction (PCR) using primers and amplification conditions described previously [23, 24]. Ten microlitres of the PCR products was resolved by electrophoresis on a 2% agarose gel and sized against 1000-basepair (bp) molecular weight marker (New England Biolabs, Beverly, MA). Primers sequences and PCR conditions for the nested PCR strategy were as previously described [23, 24]. Each *P. falciparum* infection was characterized on the basis of the fragment size of the PCR products for each locus and determining size of the alleles of MSP-1, MSP-2 and GLURP. Infections were defined as polyclonal if parasites from the same patient showed more than one allele on one or more genes. If an isolate had one allele at each of the 3 loci, the clone was taken to be one. Extracted parasite DNA was not quantified but intensity of parasite DNA band and time of maximal intensity was noted on electrophoresis in each patient. Because qPCR was not performed, internal controls like human housekeeping genes were not used. However, DNA concentration in different time point samples were quantified using a NanoDrop 2000c. Equal volumes of DNA were used in a 25 μL final volume of PCR reaction. Parasite DNA clearance time was defined as time from commencement of treatment until there was no detectable parasite DNA by genotyping.

#### Haematological evaluation

Capillary blood collected before treatment and during follow-up was used to measure haematocrit using a microhaematocrit tube and microcentrifuge (Hawksley, Lancing, UK). Anaemia was defined as a haematocrit < 30% [6, 25]. Late-appearing anaemia (LAA)] was defined as anaemia occurring after 2 weeks of starting treatment [7]. A diagnosis of late-appearing anaemia was made if the following criteria were met following initiation of artemisinin-based combination treatments: clearance of parasitaemia and other symptoms within 1 week, haematocrit ≥ 30% at 1 and/or 2 weeks, a fall in haematocrit to < 30% occurring at 3–6 weeks, absence of concomitant illness at 1–6 weeks, and absence of asexual parasitaemia by both microscopy and PCR at 3–6 weeks [7]. Anaemia recovery time (in anaemic patients at presentation) was defined as time elapsing from start of drug administration to attainment of a haematocrit value ≥ 30% [26, 27]. Fall in haematocrit (FIH) per 1000 asexual parasites cleared from peripheral blood following treatment [FIH/1000 asexual parasites cpb] was defined as relative difference in haematocrit at baseline (pre-treatment) and the first 1 or 2 days after treatment began as numerator, and the corresponding relative difference in parasitaemia as the denominator, and expressing it per 1000 asexual parasites cleared from peripheral blood [28].

#### Evaluation of response to treatment

Response to drug treatment was assessed using a modified version of the World Health Organization in vivo clinical classification criteria [29]. The clinical classification system consisted of the following categories of response: adequate clinical and parasitological response (ACPR), late parasitological failure (LPF), late clinical failure (LCF), and early treatment failure (ETF). The primary outcomes were the 42-day uncorrected and PCR-corrected efficacy. The secondary outcomes were the fever clearance time, parasite clearance time and recovery from malaria-associated anaemia.

The cure rates on days 28 and 42 were adjusted on the basis of the PCR (polymerase chain reaction) genotyping results of paired samples of patients with recurrent parasitaemia after day 7 of starting treatment as previously described [23]. Fever clearance time (FCT) in patients with presenting body temperature ≥ 37.5 °C was defined as time elapsing from start of treatment until temperature fell below 37.5 °C and remained so for at least 48 h. Parasite clearance time (PCT) was defined as time elapsing from start of drug administration until there was no patent parasitaemia for at least 72 h. Asexual parasite reduction ratio (PRR) [30] was defined as the ratio of day 0/day 2 parasitaemia (and for convenience, referred to as PRR_D2_). Asexual parasite reduction ratio on day 1 (PRR_D1_) was defined as the ratio of day 0/day 1 parasitaemia.

#### Kinetics of release of asexual parasites into and of their disposition from peripheral blood in children with ERAP, and of the disposition of asexual parasites in children without ERAP

In order to characterize the release of asexual parasites into peripheral blood from ‘deep tissue’, a graphic procedure similar to that for testing whether drug absorption is a first–order process, and if so, to determine the absorption half-life - the method of residuals, was adapted [31]. Asexual parasites in deep tissue was assumed to be the administered ‘drug’ and the rate of increase and decrease in peripheral blood asexual parasites, the ‘drug’ absorbed into circulation as follows: in each patient, on a semi-log plot of parasite density (concentration) versus time, the linear portion of the decline phase was back extrapolated. The concentration along the extrapolated line was denoted as $\overleftarrow{C}$. The observed parasite concentration during the rising phase, denoted as *C*, was subtracted from the corresponding extrapolated value at each time point, that is, $\overleftarrow{C}- C$ (the residuals). The residuals $\left(\overleftarrow{C}- C\right)$ was plotted against time on the same semi-logarithmic graph paper. Release half-time (the equivalent of absorption half-time) was determined from this plot. Rate constant for release (K_erap_) [the equivalent of absorption rate constant] was calculated as 0.693/t_½erap_. Lag time for release (the equivalent of time between drug administration and onset of absorption) was the time when the plot of $\overleftarrow{C}- C$ meets $\overleftarrow{C}$. This time was assumed to be the interval between time of administration of first dose of ACTs and the onset of release of asexual parasites from deep tissue into peripheral blood (see reference [31] for the underlying basis of the method of residuals and its equations). Areas under curve (AUC) of the plot of parasitaemia versus time, and of the plot of clone number versus time were estimated by trapezoidal method [31]. Kinetics of elimination of parasitaemia were evaluated as previously described [32, 33].

### Statistical analysis

Data were analyzed using version 6 of Epi-Info software [34] and the statistical program SPSS for Windows version 20.0. [35]. Variables considered in the analysis were related to the densities of *P. falciparum* asexual and sexual forms. Proportions were compared by calculating *χ* ^2^ using Yates’ correction, Fisher’s exact or Mantel Haenszel tests. Normally distributed, continuous data were compared by Student’s *t* test and analysis of variance (ANOVA). Data not conforming to a normal distribution were compared by the Mann–Whitney U tests and the Kruskal Wallis tests. The relationship between two variables that are continuous and normally distributed, and those that are discrete and not normally distributed were evaluated by Pearson correlation coefficient and Spearman’s rank correlation coefficient, respectively. A stepwise multiple logistic regression model was used to test the association between early rising asexual parasitaemia and factors that were significant at univariate analysis: parasitaemia <100,000/uL, parasitaemia 1 day after treatment began, and parasite reduction ratio <10^4^ 1 day after treatment began. Because the study was conducted over a period of 7 years, time in years since the commencement of the study was included as a dichotomous covariate in the model for early rising asexual parasitaemia. *P* values of <0.05 were taken to indicate significant differences. Data were double entered serially using patients’ codes and were only analyzed at the end of the study.

## Results

### Characteristics of patients enrolled in the study and treatment outcomes

The characteristics of children enrolled in the study are shown in Table 2. These characteristics are similar in children with and without ERAP. However, children with ERAP had significantly lower enrolment geometric mean parasitaemia [52,392 μL^−1^ (range 2,220–536,912, *n* = 205) *versus* 61,933 μL^−1^ (range 1,800–1,096,636, *n* = 211), respectively, *P* = 0.01] and proportion with a parasitaemia >100,000 μL^−1^ (19.6% *versus* 40.3%, *P* < 0.0001).

**Table 2:** Clinical, parasitological and other parameters at enrolment in children with or without early rising asexual parasitaemia following artemisinin-based combination treatments

	Early rising asexual parasitaemia			*P* value
	Yes (*n* = 205)	No (*n* = 211)	ALL (*n* = 416)	
Gender (M/F [% female])	116/89 [43]	117/94 [45]	233/183 [44]	0.89
Age (year)				
Mean	7.8	7.5	7.6	0.44
95% CI	7.3–8.2	7.1–8	7.3–8	
No. < 5 years	36	48	84	0.23
Duration of illness (day)				
Mean	2.8	2.8	2.8	0.52
95% CI	2.6–2.9	2.6–3	2.7–2.9	
Temperature (°C)				
Mean	38.3	38.1	38.2	0.51
95% CI	38.1–38.5	37.9–38.3	38.1–32.7	
No. ≥ 37.5 °C	154	141	295	0.06
No. ≥ 40 °C	15	15	30	0.94
Haematocrit (%)				
Mean	32.1	32.4	32.3	0.43
95% CI	31.5–32.7	31.8–33	31.8–32.7	
No. <30%	49	47	96	0.78
Parasitaemia (μL^−1^)				
Geometric mean	52,392	61,933	57,032	0.01
Range	2,220–536,912	1,800–1,096,636	1,800–1,096,636	
No. ≥ 100,000 μL^−1^	40	85	125	<0.0001
No. ≥ 250,000 μL^−1^	12	24	36	0.045

*CI* confidence interval

### Frequency of early rising asexual parasitaemia

**Fig. 1:**
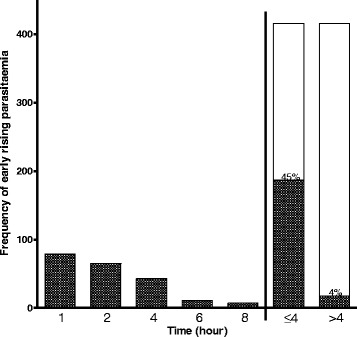
Frequency and time distribution of children with early rising asexual parasitaemia following artemisinin-based combination treatments

Two hundred and five of 416 children (49%) had ERAP and it occurred with similar frequencies in those treated with artesunate-amodiaquine, artemether-lumefantrine or dihydroartemisinin-piperaquine [107 of 240 (45%) *versus* 64 of 120 (53%) *versus* 34 of 56 (61%), respectively, *P* = 0.054]. ERAP was significantly more frequent in the first 4 h following initiation of treatment compared to > 4 h after start of treatment (187 of 416 *versus* 18 of 416 (*P* < 0.0001) (Fig. 1).

### Factors contributing to early rising asexual parasitaemia

Factors at presentation associated with ERAP following ACTs are presented in Table 3. In a univariate analysis, an enrolment parasitaemia <100,000 μL^−1^, parasite positivity on day 1 and parasite reduction ratio <10^4^ one day after treatment began were related to ERAP. Gender, age, duration of illness, temperature at presentation, fever 1 day after treatment began, anaemia at presentation and 1 day after treatment began, FIH/1000 asexual parasites cpb, fever clearance time, parasite reduction ratio 2 days after treatment began, and year and season of enrolment were not related to ERAP. In a multivariate analysis, an enrolment parasitaemia <100,000 μL^−1^ and parasite positivity 1 day after treatment began were independent predictors of ERAP.

**Table 3:** Risk factors for early rising asexual parasitaemia in malarious children following artemisinin-based combination treatments

Variable	Total no.	No. with early rising asexual parasitaemia	OR (95% CI)	*P* value	AOR (95% CI)	*P* value
Gender						
Female	209	103	1			
Male	207	102	1.0 (0.7–1.5)	1.0	-	-
Age (years)						
≥ 5	332	169	1			
< 5	84	36	0.8 (0.4–1.2)	0.19	-	-
Duration of illness (days)						
≤ 2 days	129	61	1			
> 2 days	226	117	1.2 (0.8–1.8)	0.42	-	-
Enrolment body temperature						
≤ 38 °C	230	123	1			
> 38 °C	186	82	1.5 (1.1–2.2)	0.06	-	-
Fever on day 1						
Absent	383	187	1			
Present	30	16	1.2 (0.6–2.5)	0.63	-	-
Haematocrit at presentation (day 0)						
≥ 30%	320	156	1			
< 30%	96	49	1.1 (0.7–1.7)	0.69	-	-
Haematocrit on day1						
≥ 30%	277	110	1			
< 30%	121	66	1.5 (0.9–2.2)	0.09	-	-
FIH						
> 0.05	67	29	1			
≤ 0.05	113	58	0.7 (0.4–1.3)	0.37	-	-
Parasitaemia (μL^−1^)						
≥ 100,000	125	40	1		1	
< 100,000	291	165	2.8 (1.8–4.3)	<0.0001	3.3 (2.1–5.3)	<0.0001
Parasitaemia on day 1						
Absent	325	144	1		1	
Present	91	61	2.6 (1.6–4.2)	<0.0001	3.8 (1.6–8.9)	0.002
PRR_D1_						
≥ 10^4^	302	134	1		1	
< 10^4^	114	71	2.1 (1.3–3.2)	0.001	0.9 (0.4–1.7)	0.52
PRR_D2_						
≥ 10^4^	378	187	1			
< 10^4^	38	18	0.9 (0.5–1.8)	0.8	-	-
Fever clearance time						
≤ 1 day	269	140	1			
> 1 day	26	14	1.2 (0.5–2.7)	0.63	-	-
Season of enrolment						
Dry	97	44	1			
Wet	319	161	1.2 (0.8–1.9)	0.38	-	-
Year of enrolment						
≤ 2010	254	119	1			
> 2010	162	86	1.3 (0.9–1.9)	0.21	-	-
Treatment						
Artesunate-amodiaquine	240	107	1			
Artemether-lumefantrine	120	64	1.4 (0.9–2.2)	0.12	-	-
Dihydroartemisinin-piperaquine	56	34	1.9 (1.1–3.5)	0.03		

*OR* odd ratio, *AOR* adjusted odd ratio, *CI* confidence interval, *FIH* fall in haematocrit per 1000 asexual parasite cleared from peripheral, *PRR*_D1_ parasite reduction ratio 1 day after treatment began, *PRR*_D2_ parasite reduction ratio 2 days after treatment began

### Time-course of parasitaemia in patients with or without early rising asexual parasitaemia

**Fig. 2:**
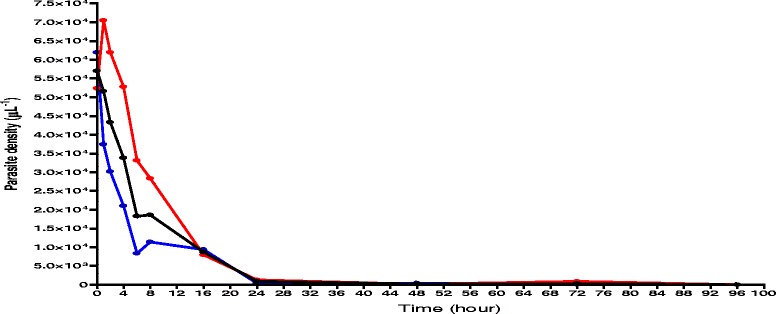
Time-course of parasitaemia in malarious children with early rising asexual parasitaemia (*red line*), without early rising asexual parasitaemia (*blue line*), and in all children (*black line*) following treatment with artesunate-amodiaquine, artemether-lumefantrine or dihydroartemisinin-piperaquine

The time-course of parasitaemia in children with or without ERAP is shown in Fig. 2.

- * Increase in pre-treatment parasitaemia after first dose of ACTs*
  Overall, mean maximum increase in parasitaemia over baseline (pre-treatment) was 96% (95% CI 73.4–118.6, *n* = 226) in all children. In children with ERAP, overall, mean maximum increase in pre-treatment parasitaemia was 105.6% (95% CI 81–130.1, *n* = 205), and it was similar with all 3 treatments [92.5% (95% CI 62.2–122.4, *n* = 107) *versus* 125.5% (95% CI 69.9–181.2, *n* = 64) *versus* 109.3% (95% CI 56.8–161.1, *n* = 34) in artesunate-amodiaquine, artemether-lumefantrine and dihydroartemisinin-piperaquine treatment groups, respectively, *P* = 0.5]. In children without ERAP mean maximum increase in parasitaemia was 2.5% (95% CI 1.9–3.1, *n* = 21). At presentation, geometric mean parasitaemia was significantly higher in children without ERAP compared with those with ERAP [61,933 μL^−1^(range 1,800–1,096,636) *versus* 52,392 μL^−1^ (range 2,220–536,912), *P* = 0.01]. However at other times following the first dose of ACTs, geometric mean parasitaemia was significantly higher in children with ERAP compared with those without [geometric mean 70,313 μL^−1^ (range 2,136 – 763,500) *versus* 37,425 μL^−1^ (range 177 – 473,059), *P* < 0.0001; geometric mean 61,965 μL^−1^ (range 2,976 – 838,500) *versus* 29,719 μL^−1^ (range 60 – 358,800), *P* < 0.0001; geometric mean 52,778 μL^−1^ (range 210 – 785,610) *versus* 20,873 μL^−1^ (range 30 – 326,400), *P* < 0.0001; geometric mean 33,660 μL^−1^ (range 980 – 53,100) *versus* 8,834 μL^−1^ (60 – 141,556), *P* < 0.0001; geometric mean 28,360 μL^−1^ (range 36 – 616,119) *versus* 11,406 μL^−1^ (range 23 – 367,647), *P* = 0.002 at 1, 2, 4, 6 and 8 h, respectively].
- * Time to peak parasitaemia after first dose of ACTs*
  Overall, time to peak parasitaemia in all children after the first dose was 1.3 h (95% CI 1.1–1.5, *n* = 226). In children with ERAP, mean peak asexual parasitaemia occurred at 2.5 h (95% CI 2.2–2.7, *n* = 205), and it was similar with all 3 treatments [2.3 h (95% CI 2–2.7, *n* = 107) *versus* 2.5 h (95% CI 2–3, *n* = 64) *versus* 2.8 h (95% CI 2.2–3.5, *n* = 34) in artesunate-amodiaquine, artemether-lumefantrine and dihydroartemisinin-piperaquine treatment groups, respectively, *P* = 0.39]. In children without ERAP, time to peak parasitaemia occurred at 0.2 h (95% CI 0.1–0.3, *n* = 21), and it was significantly lower than in those with ERAP (*P* < 0.0001).
- * Parasite and fever clearance*
  Overall, mean parasite clearance time was 29 h (95% CI 27.2–30.8, *n* = 416). Parasite clearance was significantly faster in children without compared to those with ERAP [27.9 h (95% CI 26.5–29.3, *n* = 211) versus 32.1 h (95% CI 30.2–33.9, *n* = 205), respectively, *P* < 0.0001]. In those with ERAP, parasite clearance was significantly faster in artesunate-amodiaquine-treated children compared with artemether-lumefantrine- or dihydroartemisinin-piperaquine-treated children [28.1 h (95% CI 26.7–29.5, *n* = 240) *versus* 30.6 h (95% CI 28.4–32.8, *n* = 120) *versus* 36.4 h (95% CI 32.5–40.3, *n* = 56), respectively, *P* = 0.04]. In those without ERAP, parasite clearance was significantly slower in dihydroartemisinin-piperaquine-treated children compared with artesunate-amodiaquine- or artemether-lumefantrine-treated children [34.6 h (95% CI 28.6–41.2, *n* = 22) *versus* 27.4 h (95% CI 25.7–29.2, *n* = 133) *versus* 26.1 h (95% CI 24.3–28, *n* = 56), respectively, *P* = 0.002]. Overall, PCR corrected ACPR on days 28–42 was 97.8% (95% CI 95–100) and it was similar in children with and without ERAP [97.3% (95% CI 94.1–100) *versus* 98.2% (95% CI 95.4–100), respectively; *P* = 0.38]. No child had early treatment failure (ETF). Late parasitological failure (LPF) occurred in 13 children (8 of 205 children *versus* 5 of 211 children in those with and without ERAP, respectively). There was no significant difference in the proportions of children with late parasitological failure in the two groups (*P* = 0.37). Overall parasite positivity on day 3 was 3 of 416 and it similar in children with and without ERAP (2 of 205 *versus* 1 of 211, respectively, *P* = 0.62). Overall, mean fever clearance time was 1.1 day (95% CI 1.06–1.1, *n* = 295). Fever clearance was similar in children with or without ERAP [1.1 day (95% CI 1–1.2, *n* = 154) *versus* 1.1 day (95% CI 1–1.1, *n* = 141), respectively, *P* = 0.62].
- * Parasite reduction ratio*
  Parasite reduction ratio 1 and 2 days after treatment began was significantly higher in children without compared with those with ERAP [2.8 × 10^4^ (7.8 × 10^0^–1.1 × 10^6^) *versus* 6.2 × 10^3^ (5.6 × 10^−1^–5.4 × 10^5^), respectively, *P* < 0.0001 and 5.7 × 10^4^ (1.6 × 10^1^–1.1 × 10^6^) *versus* 4.6 × 10^3^ (3.5 × 10^1^–5.4 × 10^5^), respectively, *P* < 0.005]. In children with ERAP, parasite reduction ratio 1 day after treatment began was significantly lower in dihydroartemisinin-piperaquine-treated children compared with artesunate-amodiaquine- or artemether-lumefantrine-treated children [1.6 × 10^3^ (5.6 × 10^−1^–1.2 × 10^5^) *versus* 1.3 × 10^4^ (2.8 × 10^0^–4.7 × 10^5^) *versus* [3.7 × 10^4^ (8.0 × 10^−1^–5.4 × 10^5^), respectively, *P* = 0.01]. However, parasite reduction ratio 2 days after treatment began was similar in all 3 treatment groups [4.8 × 10^4^ (1.4 × 10^2^–4.7 × 10^5^) *versus* 5.4 × 10^4^ (1.8 × 10^3^–5.4 × 10^5^) *versus* [4.6 × 10^4^(3.5 × 10^1^–4.2 × 10^5^), respectively, *P* = 0.09]. *Post hoc* comparison showed that parasite reduction ratio 2 days after treatment began was significantly higher in artemether-lumefantrine-treated children compared with dihydroartemisinin-piperaquine-treated children (*P* = 0.03).

### Kinetics of release of asexual parasites in children with ERAP

**Fig. 3:**
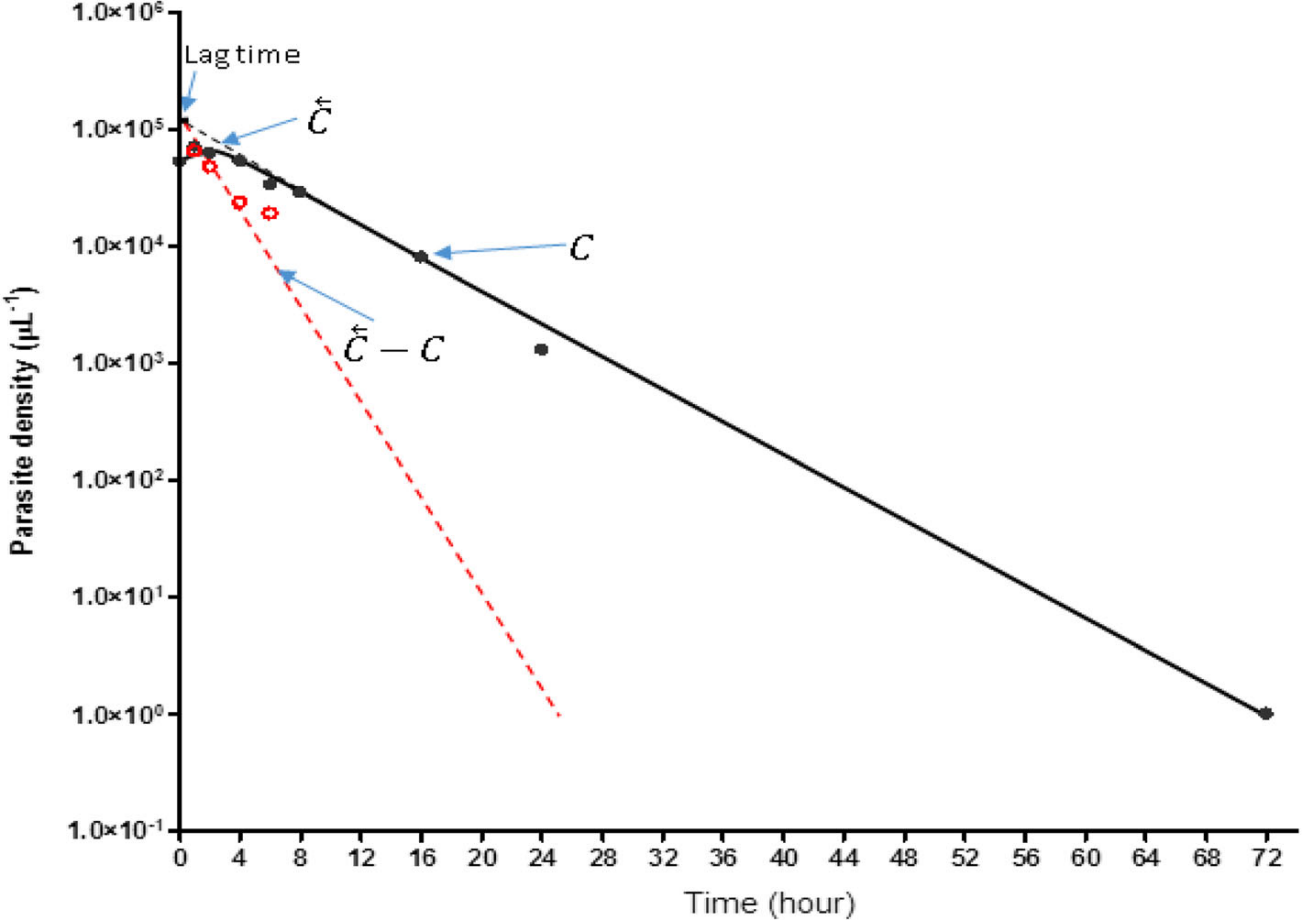
Estimates of lag time, half-time and rate constant of release of asexual parasitaemia by the method of residuals following a first dose of artemisinin-based combination treatments of falciparum malaria (mean lag time, half-time and rate constant of release were 0.2 h, 1 h and 0.9 h^−1^, respectively)

Data for evaluation of estimates of lag time, half-time and the corresponding release rate constant were available in all 205 children with ERAP. Figure 3 is a semi-logarithmic plot of parasitaemia *versus* time by the method of residuals in all 205 children with ERAP.

- * Lag time*: Overall, mean lag time was 0.2 h (95% CI 0.2-0.3, range 0.02-0.9) and it was similar with all 3 treatments: [0.2 h (95% CI 0.2–0.3, range 0.02–0.9, *n* = 107) *versus* 0.2 h (95% CI 0.2–0.3, range 0.02–0.8, *n* = 64) *versus* 0.2 h (95% CI 0.2–0.3, range 0.02–0.7, *n* = 34), respectively for AA, AL, and DHP, *P* = 1.0].
- * Half time*: Overall, mean half-time of release of parasites into peripheral circulation was 1 h (95% CI 0.9–1.1, range 0.1–4.9) and it was similar with all 3 treatments: [1 h (95% CI 0.9–1.1, range 0.1–4.9, *n* = 107) *versus* 1.2 h (95% CI 0.9–1.4, range 0.3–4.4, *n* = 64) *versus* 1 h (95% CI 0.8–1.2, range 0.5–3.6, *n* = 34), respectively for AA, AL, and DHP, *P* = 0.34].
- * Rate constant of release*: Overall, mean rate constant for release of parasites into peripheral circulation was 0.9 h^−1^ (95% CI 0.8–1, range 0.1–5.6) and it was similar with all 3 treatments: [0.9 h^−1^ (95% CI 0.8–1.1, range 0.1–5.6, *n* = 107) *versus* 0.8 h^−1^(95% CI 0.7–0.9, range 0.2–2.4, *n* = 64) *versus* 0.9 h^−1^ (95% CI 0.7–0.9, range 0.2–1.5, *n* = 34), respectively for AA, AL, and DHP, *P* = 0.45].
- * Factors influencing kinetics of the release of asexual parasitaemia during early rising asexual parasitaemia*: Table 4 shows the clinical and parasitological parameters that can influence the kinetics of release of asexual parasitaemia in children with early rising asexual parasitaemia. Of these parameters, only the parasite clearance time significantly affected both the lag time and the half-time of release of asexual parasitaemia. With respect to the influence of parasite clearance, there is a reciprocal relationship between lag time and half-time of release if parasite clearance time was ≤ 1 day.

**Table 4:** Factors influencing the kinetics of release of asexual parasites in children with early rising asexual parasitaemia

Variables		Lag time [Mean (95% CI)] (h)	*P* value	t_½rls_ [Mean (95% CI)] (h)	*P* value
Age	<5 years (*n* = 36) *v*	0.3 (0.2–0.3) *v*	0.17	1 (0.7–1.2) *v*	0.42
	≥5 years (*n* = 169)	0.2 (0.18–0.2)		1 (0.9–1.1)	
Gender	Male (*n* = 116) *v*	0.2 (0.17–0.2) *v*	0.21	1.1 (1–1.3) *v*	0.02
	Female (*n* = 89)	0.2 (0.2–0.3)		0.9 (0.8–1)	
Duration of illness	≤2 days (*n* = 61) *v*	0.2 (0.2–0.3) *v*	0.38	0.9 (0.8–1) *v*	0.02
	>2 days (*n* = 117)	0.2 (0.2–0.3)		1.1 (0.9–1.2)	
Enrolment parasitaemia	≤100000 μL^−1^ (*n* = 40) *v*	0.2 (0.1–0.3) *v*	0.43	1 (0.8–1.3) *v*	0.98
	>100000 μL^−1^ (*n* = 165)	0.2 (0.2–0.3)		1 (0.9–1.1)	
Enrolment temperature	≤37.4 °C (*n* = 51) *v*	0.3 (0.2–0.3) *v*	0.21	1 (0.7–1.2) *v*	0.4
	>37.4 °C (*n* = 154)	0.2 (0.18–0.2)		1 (0.9–1.1)	
Haematocrit	<30% (*n* = 49) *v*	0.2 (0.2–0.3) *v*	0.54	1.1 (0.9–1.3) *v*	0.22
	≥30% (*n* = 156)	0.2 (0.2–0.3)		1 (0.9–1.1)	
Fever clearance time	≤1 days (*n* = 140) *v*	0.2 (0.2–0.3) *v*	0.33	1 (0.9–1.1) *v*	0.43
	>1 days (*n* = 14)	0.2 (0.1–0.2)		0.9 (0.7–1.1)	
Parasite clearance time	≤1 days (*n* = 144) *v*	0.25 (0.21–0.28) *v*	0.01	0.8 (0.7 – 0.8) *v*	<0.0001
	>1 days (*n* = 61)	0.18 (0.14–0.21)		1.6 (1.4–1.8)	

*t*_*½rls*_ half-time of release of asexual parasitaemia into peripheral blood, *v* versus

### Kinetics of the disposition of parasitaemia in children with or without early rising asexual parasitaemia

**Fig. 4:**
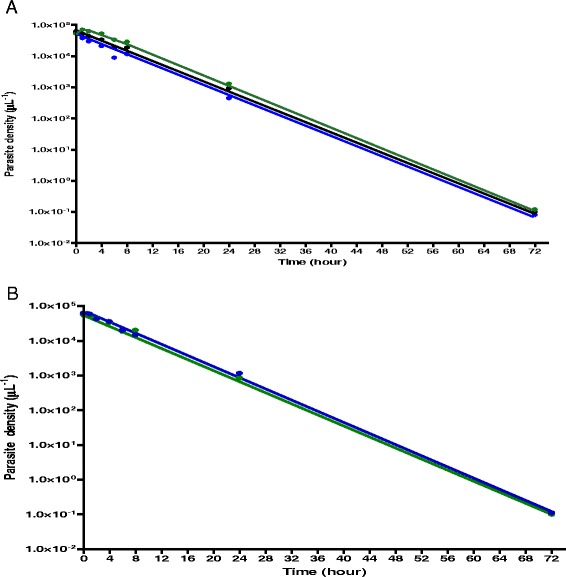
Semilogarithmic plots of asexual parasitaemia *versus* time (**a**) in all children (*black line*), children with (*green line*) and without (*blue line*) early rising asexual parasitaemia, and (**b**) in anaemic (*blue line*) and non-anaemic (*green line*) children treated with artesunate-amodiaquine, artemether-lumefantrine or dihydroartemisinin-piperaquine

Overall, geometric mean AUC from initiation of treatment to clearance of parasitaemia in all children was 5.4 × 10^5^ μL^−1^.h (95%CI 9.0 × 10^5^–1.1 × 10^6^, range 2.7 × 10^3^–1.1 × 10^7^). Geometric mean AUCs at 0–4, 0–8, 0–24 and 0–48 h after first dose were significantly higher in children with early rising asexual parasitaemia compared with children without early rising asexual parasitaemia (Table 5, Fig. 3). Overall, declines from peak parasitaemias were monoexponential in children with or without ERAP (Fig. 4). Overall estimated half-time was 1.2 h (95% CI 1.1–1.2). Mean estimated half-times were similar in children with and without ERAP [1.2 h (95% CI 1.1–1.3) *versus* 1.2 h (95% CI 1.1–1.3), respectively; *P* = 0.6] and in all treatment groups [1.2 h (95% CI 1.1–1.2, *n* = 240) *versus* 1.2 h (95% CI 1.1–1.2, *n* = 120) *versus* 1.3 (95% CI 1.1–1.4, *n* = 56), *P* = 0.61] in artesunate-amodiaquine-, artemether-lumefantrine- and dihydroartemisinin-piperaquine-treated children, respectively. Mean estimated half-times were also similar in anaemic and non-anaemic children [1.2 h (95% CI 1.1–1.3, *n* = 96) *versus* 1.2 h (95% CI 1.1–1.2, *n* = 320), respectively; *P* = 0.49, Fig. 4]. In children with ERAP, mean estimated half-times were similar with all 3 treatments [1.2 h (95% CI 1.1–1.2, *n* = 107) versus 1.2 h (95% CI 1.1–1.3, *n* = 64) versus 1.3 (95% CI 1.1–1.4, *n* = 34); *P* = 0.44] in artesunate-amodiaquine-, artemether-lumefantrine- and dihydroartemisinin-piperaquine-treated children, respectively. Similarly, in children without ERAP, estimated half-times were similar with all 3 treatments [1.2 h (95% CI 1.1–1.3, *n* = 133) versus 1.2 h (95% CI 1–1.2, *n* = 56) versus 1.3 (95% CI 1.1–1.5, *n* = 22); *P* = 0.64] in artesunate-amodiaquine-, artemether-lumefantrine- and dihydroartemisinin-piperaquine-treated children, respectively. The other pharmacokinetic parameters are summarized in Table 5.

**Table 5:** Parameters for disposition of asexual parasitaemia in children with or without early rising asexual parasitaemia following artemisinin-based combination treatments

Parameters	Early rising asexual parasitaemia			*P* value
	Yes (*n* = 205)	No (*n* = 211)	All (*n* = 416)	
C_maxpd_ (μL^−1^)				
Geometric mean	91,502	61,639	74,887	0.01
Range	3,465-838,500	1,800-1,096,636	1,800-1,096,636	
T_maxpd_ (h)				
Mean	2.6	0.2	1.4	<0.0001
95% CI	2.3–2.9	0.1–0.3	1.2–1.6	
AUC_pd_ (μL^−1^.h)				<0.0001
0-4 h (Geometric mean)	2.6 × 10^5^	1.5 × 10^5^	2.0 × 10^5^	
Range	1.0 × 10^4^–3.1 × 10^6^	1.6 × 10^3^–2.2 × 10^6^	1.6 × 10^3^–3.1 × 10^6^	
0–8 h (Geometric mean)	5.3 × 10^5^	2.7 × 10^5^	3.9 × 10^5^	0.001
Range	2.0 × 10^4^–5.9 × 10^6^	2.2 × 10^3^–2.4 × 10^6^	2.2 × 10^3^–5.9 × 10^6^	
0–24 h (Geometric mean)	7.8 × 10^5^	3.6 × 10^5^	5.3 × 10^5^	<0.0001
Range	2.9 × 10^4^–1.1 × 10^7^	2.7 × 10^3^–4.1 × 10^6^	2.7 × 10^3^–1.1 × 10^7^	
0–48 h (Geometric mean)	7.9 × 10^5^	3.7 × 10^5^	5.4 × 10^5^	<0.0001
Range	2.9 × 10^4^–1.1 × 10^7^	2.7 × 10^3^–4.5 × 10^6^	2.7 × 10^3^–1.1 × 10^7^	
0–∞ (Geometric mean)	8.0 × 10^5^	3.7 × 10^5^	5.4 × 10^5^	<0.0001
Range	2.9 × 10^4^–1.1 × 10^7^	2.7 × 10^3^–4.5 × 10^6^	2.7 × 10^3^–1.1 × 10^7^	
CL_Bpd_ (Lh^−1^)				
Mean	0.1	0.4	0.3	<0.0001
95% CI	0.1–0.2	0.3–0.4	0.2–0.3	
Elimination half-time (h)				
Mean	1.2	1.2	1.2	0.6
95% CI	1.1–1.3	1.1–1.2	1.1–1.2	
Elimination rate constant (h^−1^)				
Mean	0.6	0.6	0.6	0.9
95% CI	0.6–0.7	0.6–0.7	0.6–0.7	

*h* hour, *C*_*maxpd*_ maximum asexual parasite density (concentration), *T*_*maxpd*_ time to reach maximum asexual parasite density (concentration), *CL*_*Bpd*_ volume of blood cleared of parasitaemia per unit of time, *AUC*_*pd*_ area under curve of plot of parasite density (parasitaemia) *versus* time

### Stages of sexual and asexual parasitaemias in peripheral blood

**Fig. 5:**
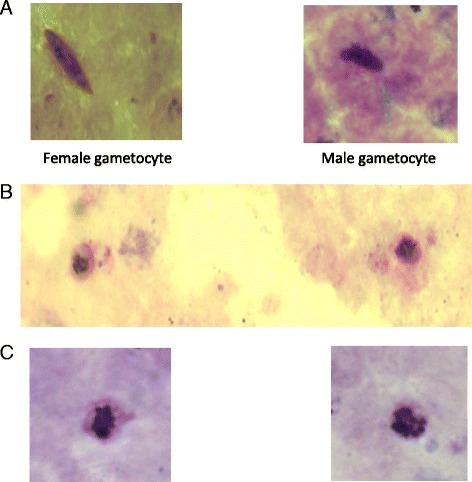
Light micrograph of *Plasmodium falciparum* in the first 8 h after a first dose of artemisinin-based combination treatments*.* Panel **a** shows female and male gametocytes, Panel **b** shows immature gametocytes, and Panel **c** shows schizonts found in peripheral blood of children with early rising asexual parasitaemia

- * Before treatment*
  Before treatment, gametocytaemia was detected in peripheral blood of 12 children (3%): 6 children each with or without early rising asexual parasitaemia. Only mature gametocytes (Stages IV and V) were detectable in peripheral blood. Ring forms of varying stages were found in the peripheral blood of all children. Late sequestering trophozoites and schizonts were not found in peripheral blood of any child.
- * Following first dose of ACTs*
  In the first 8 h following treatment, in children without ERAP, different stages of ring forms were found in peripheral blood of all children but schizonts were found in peripheral blood of 1 child. However, young gametocytes were not found in peripheral blood. In children who subsequently developed ERAP, different stages of ring forms were found in peripheral blood of all children. However, schizonts were found in peripheral blood films of 4 children and young gametocytes in peripheral blood films of another 8 children (Fig. 5). After 2 weeks following treatment, gametocytes were not found in peripheral blood of any child.

### Molecular genotyping

Table 6 shows the features of the 170 children (85 pairs) with and without ERAP who were matched for clinical and parasitological parameters at presentation. Asexual parasitaemia was significantly higher in children with ERAP compared to those without from 1 to 8 h after initiation of treatment (P ≤ 0.02 at all times, Table 6). However, parasite clearance time determined by microscopy was similar in children with and without ERAP.

**Table 6:** Features of children with or without early rising asexual parasitaemia matched for gender, enrolment parasitaemia, treatment and same day presentation who were treated with artesunate-amodiaquine, artemether-lumefantrine or dihydroartemisinin-piperaquine

	Early rising asexual parasitaemia			*P* value
	Yes (*n* = 85)	No (*n* = 85)	ALL (*n* = 170)	
Temperature (°C)				
Mean	38.3	38	38.1	0.07
95% CI	38.1–38.6	37.7–38.3	37.9–38.3	
Fever on day 1	9	5	14	0.27
Fever clearance time (day)				
Mean	1.1 (*n* = 66)	1.1 (*n* = 52)	1.1 (*n* = 118)	0.59
95% CI	1–1.2	1–1.2	1–1.1	
Geometric mean parasitaemia (μL^−1^)				
At enrolment (0 h)	61,165	59,853	60,505	0.96
Range	2,928–536,912	2,000–500,000	2,000–536,912	
1 h	74,268	39,865	55,059	0.001
Range	2, 136–763,500	790–314,030	790–763,500	
2 h	71,410	30,689	46567	<0.0001
Range	2,976–838,500	284–313,980	284–838,500	
4 h	57,650	24,043	38,394	<0.0001
Range	210–785,610	339–253,304	210–785,610	
6 h	33,950	9,604	18,312	0.02
Range	980–531,000	321–133,400	321–531,000	
8 h	37,017	8,052	16,919	0.002
Range	656–616,119	106–266,400	106–616,119	
Parasite positivity				
On day 1 [%]	18 [21]	13 [15]	31 [20]	0.32
On day 3 [%]	1 [1]	1 [1]	2 [1]	1.0
PRR_D1_				
Geometric mean	1.6 × 10^4^	2.7 × 10^4^	2.1 × 10^4^	0.68
Range	6.5 × 10^−1^–5.4 × 10^5^	9.8 × 10^0^–5.0 × 10^5^	6.5 × 10^−1^–5.4 × 10^5^	
PRR_D2_				
Geometric mean	6.1 × 10^4^	5.6 × 10^4^	5.9 × 10^4^	0.78
Range	2.9 × 10^3^–5.4 × 10^5^	6.0 × 10^2^–5.0 × 10^5^	6.0 × 10^2^–5.4 × 10^5^	
Parasite clearance time (day)				
Mean	29.6	28.5	29.1	0.53
95% CI	27–32.3	26.1–30.9	27.3–30.9	
Anaemia on day 1	29	22	51	0.22
Anaemia recovery time (day)				
Mean	13.2 (*n* = 21)	10.6 (*n* = 24)	11.6 (*n* = 44)	0.25
95% CI	10.5–15.9	6.8–14.4	9.2–13.9	
No. with late-appearing anaemia	12	9	21	0.64

*H* hour, *PRR*_*D1*_ parasite reduction ratio 1 day after treatment began, *PRR*_*D2*_ parasite reduction ratio 2 days after treatment began, *CI* confidence interval

**Fig. 6:**
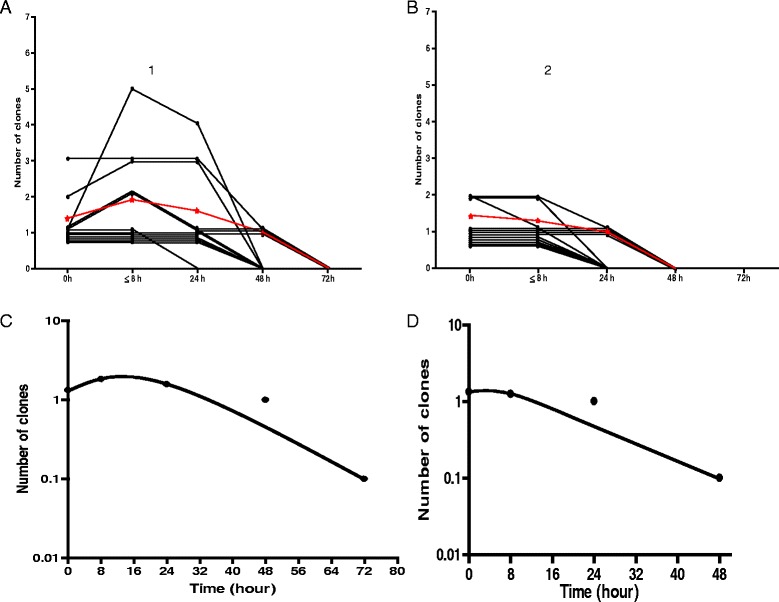
Time-course of changes in parasite DNA clones in age-, gender-, parasitaemia- and treatment-matched children with [1] and without [2] early rising asexual parasitaemia following artemisinin-based combination treatment. Panel **a** shows individual (*black lines*) and mean (*red line*) of area under curves (AUC of clone number *versus* time) in children with ERAP. Panel **b** shows individual (*black lines*) and mean (*red line*) of area under curves (AUC of clone number *versus* time) in children without ERAP. Panels **c** and **d** show semilog plots of mean clone number *versus* time in children with and without ERAP, respectively. Declines from peak clone number were monoexponential (mean estimated half-times were 3.4 h (95% CI 3.1–3.7) and 3.1 h (95% CI 2.7–3.4), respectively)
- * Time to clearance of parasite DNA clones following treatment*
  Parasite DNA clone clearance time was significantly longer in children with compared to those without early rising asexual parasitaemia [54 h (95% CI 44.5–63.5) *versus* 36 h (95% CI 28–43.9), respectively; *P* = 0.004, see Fig. 6 Panels a and b].
- * Relationship between time-course of parasitaemia and changes in intensity of parasite DNA bands*
  In general, irrespective of clone number at enrolment, DNA band intensity did not change in the first 6–8 h in children without ERAP. In children with ERAP, band intensities increased within 1–4 h following initiation of treatment and there was a significant correlation between time of maximum parasitaemia and time of maximal parasite DNA intensity (r = 0.62, *P* = 0.03). Figure 7 shows the relationship between parasite DNA band intensity, time course of parasitaemia, and parasite lag time, half-time and rate constant of release of parasitaemia in a child with ERAP and the corresponding pair without ERAP.

- * Parasite DNA clones before and after first dose of artemisinin-based combination treatments*
  Before treatment, of the 12 children with ERAP, 10 children had 1 clone infection and 1 each had 2 and 3 clone infections. In children without ERAP, 8 children had 1 clone infection and 4 had 2 clone infections. Following first dose of ACTs in children with ERAP, clone number increased in 4 children (from 1 to 2 clones in 2 children, 1 to 5 clones in 1 child and 2 to 3 clones in 1 child). In children without ERAP, clone numbers remained unchanged in 11 of 12 children. However, clone number reduced in 1 child from 2 to 1 at 4 h after first dose of ACT. The time course of changes in parasite DNA clones before and following artemisinin-based combination treatments are shown in Fig. 6.

**Fig. 7:**
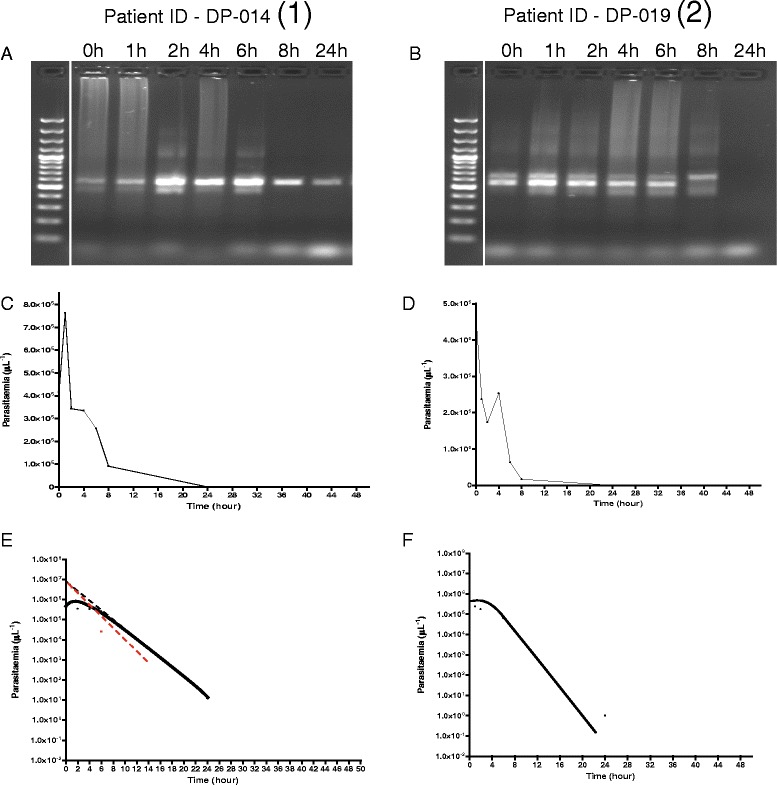
Features of molecular, time course, and release kinetics of asexual parasitaemia in age-, pre-treatment parasitaemia- and same treatment-matched malarious children with (1) and without (2) early rising asexual parasitaemia after first dose of dihydroartemisinin-piperaquine. Panel **a** shows increase in parasite clones from one to two and maximum parasite DNA band intensity 2 h post-initiation of treatment followed by decreasing intensity after 6 h. Parasite DNA clearance time was 48 h. Panel **b** shows stable number of clones (two in all) pre- and post-initiation of treatment and no change in DNA band intensity in the first 6 h followed by decrease in intensity. Parasite DNA clearance time was 24 h. Panel **c** shows peak parasitaemia at 2 h coincides with maximum DNA band intensity in Panel **a**. Panel **d** shows decreasing parasitaemia parallels DNA band intensity in Panel **b**. Panel **e** shows release kinetics estimated by method of residuals (lag time, half-time, and rate constant of 0.2 h, 0.5 h and 1.5 h^−1^, respectively, and monoexponential terminal elimination half-time of 2.3 h. Panel **f** shows only monoexponential terminal elimination half time of 1.7 h
- * Area under curve (AUC) of number of DNA clones* versus *time*
  Area under curve of DNA clone number from time of commencement of treatment till clearance was significantly higher in children with ERAP compared to those without ERAP [62.2 no.h (95% CI 36.5–87.8) *versus* 28.8 no.h (95% CI 21.5–36), *P* = 0.02) [Fig. 6]. Figure 8 shows increase in DNA clone number, area under curve of number of clone and the disposition of DNA clone in a child with ERAP and the corresponding pair without ERAP. Declines in clone number were monoexponential with similar half-times in the matched pairs of children with and without ERAP (3.4 h (95% CI 3.1–3.7) v 3.1 h (95% CI 2.7–3.4), *P* = 0.15, Figs. 6 and 8)

**Fig. 8:**
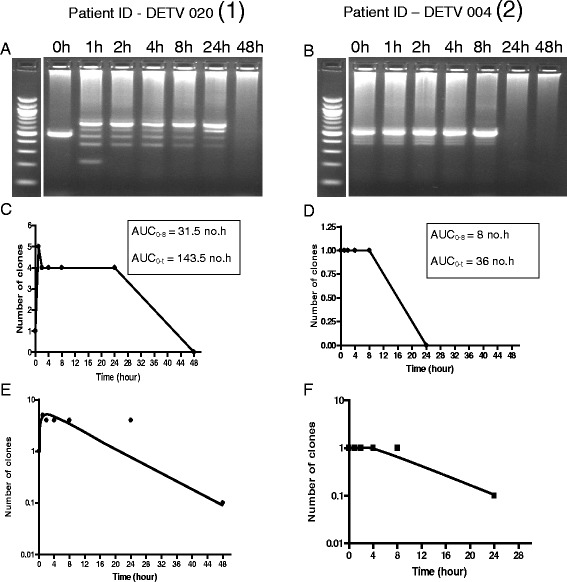
Parasite DNA clone number, area under curve of number of DNA clones *versus* time, and elimination kinetics of parasite DNA clones in age-, pre-treatment parasitaemia- and same treatment-matched malarious children with (1) and without (2) ERAP treated with artesunate-amodiaquine. Panel **a** shows increase in parasite DNA clones from one to five 1 h post-initiation of treatment. Parasite DNA clearance time was 48 h. Panel **b** shows stable number of DNA clone (one at all times) pre- and post-initiation of treatment and no change in parasite DNA clone number following treatment. Parasite DNA clearance time was 24 h. Panels **c** and **d** show area under curve of number of clones *versus* time in children with and without ERAP, respectively. Panel **e** and **f** show monoexponential terminal elimination of DNA clones in children with and without ERAP, respectively. Note: Declines in DNA clone numbers were monoexponential

### Relationship between early rising asexual parasitaemia and late-appearing anaemia

In a univariate analysis, a haematocrit <30% was not associated with early rising asexual parasitaemia (Table 3). The proportions of children with or without early rising parasitaemia who subsequently developed late-appearing anaemia were similar: 28 of 205 (14%) *versus* 24 of 211 (11%). *P* =0.48. Similarly, the proportions of children with anaemia at presentation and who subsequently developed late-appearing anaemia in children with or without early rising asexual parasitaemia were also similar: 9 of 49 (18%) *versus* 8 of 47 (17%). *P* = 0.86. When matched for age, gender, same day presentation and same treatment, the proportion of children with or without early rising parasitaemia who developed late-appearing anaemia were also similar 12 of 85 (14%) *versus* 9 of 85 (11%). *P* = 0.64 (Table 6).

### Reported adverse events

Eighty two of 205 (40%) children and 66 of 211 (31%) children with and without early rising asexual parasitaemia, respectively, reported at least 1 adverse event in the first week of starting treatment. There was no significant difference in the proportions reporting adverse events in the 2 groups (*P* = 0.08). In children with early rising asexual parasitaemia, 23 (11%), 15 (7%), 11 (5%), 2 (1%), 15 (7%), 15 (7%), 9 (4%), 11 (5%) and 3 (1%) children reported abdominal pain, fever, vomiting, weakness, headache, cough, anorexia, running nose and diarrhoea, respectively. In children without early rising asexual parasitaemia, 17 (8%), 13 (6%), 5 (2%), 1 (0.5%), 12 (6%), 16 (8%), 5 (2%), 7 (3%) and 4 (2%) children reported abdominal pain, fever, vomiting, weakness, headache, cough, anorexia, running nose and diarrhoea, respectively. There was no significant difference in the proportions reporting each of these adverse events in the children with and those without early rising asexual parasitaemia.

## Discussion

In this study, we described the clinical and parasitological features, the risk factors for, the kinetics of the release and of the disposition of asexual parasitaemia, the parasite population changes, the molecular features of and the relationship between early rising asexual parasitaemia and late-appearing anaemia following artemisinin-based combination treatments in a cohort of children resident in an endemic area. Using a definition of early rising asexual parasitaemia of ≥5% increase in baseline (enrolment) parasitaemia occurring within the first 8 h of initiating artemisinin-based combination treatments showed half of the children had significant increases in baseline parasitaemia that peaked 2.5 h after first dose. Time to peak parasitaemia was significantly longer and the peak parasitaemia significantly higher in children with compared to those without early rising asexual parasitaemia.

Of the three factors associated with early rising asexual parasitaemia (Table 3), enrolment parasitaemia <100,000/uL and parasitaemia 1 day post-treatment initiation were independent predictors of early rising asexual parasitaemia. Taken together, these factors would suggest: (i). In these children, a relatively ‘low’ baseline peripheral asexual parasitaemia may indicate a relatively ‘large’ parasite biomass in deep tissues that was, following treatment, quickly mobilized after a first dose; (ii). Mobilization resulted in significantly higher peak asexual parasitaemia compared to that in children without early rising asexual parasitaemia in the first 4–8 h post-treatment initiation (Fig. 2); (iii). The higher peripheral parasitaemia was then cleared relatively slowly. The last supports reports of slower clearance of high parasitaemias compared to lower parasitaemias by artemisinin-like drugs [33, 36]. The significant association of dihydroartemisinin-piperaquine compared with artemether-lumefantrine treatment with early rising asexual parasitaemia may in part be due to the lag time required for converting artemether to its metabolite, dihydroartemisinin. The significantly slower clearance of parasitaemia and lower parasite reduction ratio one day after treatment began in children treated with dihydroartemisinin-piperaquine may also be contributory to its significant association with early rising asexual parasitaemia (Table 3).

The rapid release of asexual parasites into peripheral blood by 12 min of initiating treatment with all 3 artemisinins coupled with the short release half-time indicate release kinetics are rapid first order processes attributable to first dose of artemisinins. Of the factors that may affect release kinetics, it would appear parasite clearance time, which showed a reciprocal relationship between lag time and half-time of release, is the most intriguing. The reason(s) is (are) unclear. However, anaemia did not affect release kinetics indicating release kinetics are not impaired in anaemic children. Evidently, there is no artemisinin resistance in *P. falciparum* in the study area ([27, 33] and as shown in the present study) and is unclear if the ‘first dose phenomenon’ will be modified by development of artemisinin resistance should this occur in the future.

Pre-treatment gametocyte carriage was low (3%) in this cohort of children. Nonetheless, young gametocytes were demonstrable after first dose only in few children with early rising asexual parasitaemia. Theoretically, their mobilization into peripheral circulation can be advantageous in two ways: exposing them to higher drug concentrations and enhancing the gametocytocidal effects of the first doses of artemisinins and their partner drugs, all of which are gametocytocidal to young gametocytes [37, 38]; and preventing further development in bone marrow to mature gametocytes which are not sensitive to killing effects of the artemisinin-based combination treatments evaluated. It has been shown that a male-biased sex ratio is more infective to mosquitoes [19], and a selective alteration of gametocyte sex ratios during early rising sexual parasitaemia (ERSP) can produce a female biased sex ratio [3, 39, 40], which is less likely to be infective to mosquitoes. However, gametocyte sex ratio changes were not evaluated in the present study because gametocytaemia was less than 10/μL, the lower threshold for estimating sex ratio [39, 40] in patients with demonstrable peripheral gametocytes. Demonstrable sequestering forms of asexual parasites (late trophozoites and schizonts) in peripheral blood also suggests mobilization from deep tissue to peripheral blood.

Overall, as expected, the area under curve of asexual parasitaemia *versus* time but not the terminal elimination half-times of parasitaemia was significantly higher in children with early rising asexual parasitaemia compared to those without. The significantly higher area under curve in the first 8–24 h post-treatment initiation indicates large peripheral asexual parasite burden in children with early rising asexual parasitaemia is largely due to a ‘first dose artemisinin phenomenon’. Also, as expected, in age-and treatment-matched children with and without early rising asexual parasitaemia, parasite clearance determined by microscopy was rapid and similar in both groups. However, parasite DNA clearance was significantly longer in children with early rising asexual parasitaemia indicating PCR is more sensitive than microscopy in detecting low peripheral parasitaemia. Taken together, the significantly higher area under curve and significantly slower clearance of parasite DNA indicate significant retention, in the peripheral blood, of asexual parasites that were likely released from deep tissue sites into peripheral blood in children with early rising asexual parasitaemia. Similarly, the significantly higher area under curve of the plot of number of clones *versus* time at 8 h or after initiation of treatment indicates significant retention of parasite clones in the peripheral blood of children with early rising asexual parasitaemia. Thus, taken together, the significant magnitude and duration of parasitaemia and parasite clones in peripheral blood during the early hours following initiation of treatment are molecular features of first dose of artemisinins and are not indicative of in vivo reduced artemisinin susceptibility in this endemic area. The monoexponential declines in clone number in children with or without early rising asexual parasitaemia who had multiple clones at initiation of treatment or following first dose indicate clone elimination is a first order process (Fig. 7). Overall, the molecular features allow the development of the ‘concept of clone retention’ (measured as area under curve) and ‘clone elimination half-time’ (determined by a monoexponential declines from peak clone number) following a first dose of an artemisinin-like drugs.

Overall, although a disadvantage of early rising asexual parasitaemia is increasing circulating parasite biomass, a distinct advantage of this first dose phenomenon is a reduced likelihood of and lower sequestering or sequestered parasite biomass. This, in addition to relatively rapid clearance of parasitaemia, should lead to reduced likelihood of progression of non-severe to severe malaria in these children because severe malaria has been attributable to parasite sequestration in vital organs [41, 42], or to release of, and imbalance between pro-inflammatory and anti-inflammatory cytokines by sequestered parasites in deep tissue [43]. In this context, controlled studies are required to evaluate the risks and consequences of progression to severe malaria in children with uncomplicated malaria who subsequently develop or did not develop early rising asexual parasitaemia following initiation of artemisinin-based combination treatments.

Although early rising asexual parasitaemia has been attributed to mobilization of parasites from deep tissues [2, 3], it could also have resulted from large numbers of schizonts bursting to release young ring forms into peripheral circulation after first dose of artemisinins. Can this also be an artemisinin effects? This is possible. The broad stage effects of artemisinins from rings aged 6 h and above [17], would act on these young rings in the first 8 h of the first dose. It is also possible that, acting by unknown mechanism, artemisinin-like drugs are more likely to cause more peripheral circulating rather than deep tissue retention of asexual parasites in children with early rising asexual parasitaemia. In this regard, studies are urgently needed on the effects of artemisinins on binding of asexual parasite-infected red blood cells to tissue proteins.

A postulate of the present study was increase mobilization of asexual parasites from deep tissue into peripheral circulation would increase the number of infected red blood cells pitted by the spleen. This process could lead to increase in number of once-infected red blood cells in peripheral circulation. The once-infected red blood cells may thereafter be destroyed 7–21 days later causing a relatively asymptomatic late-appearing anaemia following artemisinin-based combination treatments in patients with early rising asexual parasites compared with those without. This postulate was not realised in children with early rising asexual parasites compared with those without as evidenced by similar frequency of late-appearing anaemia in children with or without early rising asexual parasitaemia. Additionally, anaemia had no effect on asexual parasite release kinetics in children with early rising asexual parasitaemia. When compared with the recently described features of late-appearing anaemia [7], there are significant differences in risk factors for the two conditions. The differences are particularly striking with respect to age, presence of anaemia at enrolment or 1 day after treatment began, parasite reduction ratios, and parasite clearance times, which are diametrically opposite in these two conditions associated with artemisinin-based combination treatments (see reference [7]). Perhaps the lack of association between early rising asexual parasites and late-appearing anaemia is one of the ‘beneficial effects’ of artemisinin-like drugs. Studies are now necessary to quantify once- infected red blood cells in patients with or without early rising asexual parasites following artemisinin-based combination treatments.

There is need to justify the definition of early rising parasitaemia used in the present study. Firstly, a ≥5% increase in parasitaemia from baseline in a relatively short time frame is unlikely to be a random effect. Secondly, in a manner similar to a two-way analysis of variance, it is likely to represent a significant increase in parasitaemia from baseline in a short time frame that would result in two populations of children: those with and those without early rising asexual parasitaemia after a first dose of artemisinins. Thirdly, the lag time of 12 min after a first dose and ≥5% increase in baseline parasitaemia indicate it is a first dose artemisinin effect and it is unlikely to be due to reduced sensitivity or resistance in the parasites that are released into peripheral circulation. Fourthly, with an estimated half-life of 1 h or less, >99% of an administered first dose of oral artemisinin would have been eliminated in 8 h lending credence to this critical time frame, and attributing the response most likely but not exclusively to artemisinin-like drug action. Finally, the definition is not in agreement with in vivo measures of artemisinin resistance measured as parasite positivity rate 3 days after commencement of treatment >3% in patients with pre-treatment parasitaemia <100,000/μL or >10% of patients with detectable *P. falciparum* parasitaemia 72 h after initiation of direct observed therapy [44, 45]. The similar frequency of early rising asexual parasitaemia and non-early rising asexual parasitaemia in the population of children evaluated would suggest there is a possible genetic basis for this ‘first dose phenomenon’. Whether the genetic basis is host- or parasite-related is conjectural. More studies are now needed to explore the basis of this phenomenon.

There are limitations of the study. These include not using a quantitative method (qPCR) to assess the amount of DNA in patients samples during the various time points, in samples obtained in children with or without early rising asexual parasitaemia to justify the increased intensity observed on PCR gels and not estimating once-infected red blood cells in the postulate to establish the relationship between early rising asexual parasitaemia and late- appearing anaemia.

## Conclusion

In conclusion, early rising asexual parasitaemia is common, occurs rapidly as first order process and may be due to mobilization of parasites from deep tissue following a first dose ACTs of acute childhood falciparum malaria.

## Abbreviations

%: Percent
°C: Degree Celsius
μL: Microliter
AA: Artesunate-amodiaquine
ACTs: Artemisinin-based combination treatments
AL: Artemether-lumefantrine
AOR: Adjusted odds ratio
AUC: Area under curve
bp: Base pair
cbp: Cleared from peripheral blood
CL_Bpd_: Volume of blood cleared of parasitaemia per unit of time
C_maxpd_: Maximum parasite density
DHP: Dihydroartemisinin-piperaquine
DNA: Deoxyribonucleic acid
ERAP: Early rising asexual parasitaemia
FCT: Fever clearance time
FIH: Fall in haematocrit
g: Gram
GMPD: Geometric mean parasite density
h: Hour
HCT: Haematocrit
kg: Kilogram
L: Litre
LAA: Late-appearing anaemia
M/F: male/female
mg: milligram
OR: Odds ratio
PCR: Polymerase chain reaction
PCT: Parasite clearance time
PRR: Parasite reduction ratio
qPCR: Quantitative polymerase chain reaction
T_maxpd_: Time to reach maximum parasite density
v: versus
